# **Patellofemoral vs. total knee arthroplasty for isolated patellofemoral osteoarthritis: evidence-based recommendations from a systematic review with GRADE assessment**

**DOI:** 10.1007/s00402-026-06217-3

**Published:** 2026-02-12

**Authors:** Riccardo Sacco, Andrea Tecame, Stefaan Van Onsem, Edward Massa, Matthieu Lalevée, Paolo Adravanti

**Affiliations:** 1 Department of Orthopedic and Trauma Surgery, Centre Hospitalier Universitaire de Rouen, Rouen, France; 2 CETAPS EA3832, Research Center for Sports and Athletic Activities Transformations, University of Rouen Normandy, F-76821 Mont-Saint-Aignan, France; 3 Department of Orthopaedic and Trauma Surgery, Città di Parma Clinic, Piazzale Athos Maestri, 5, 43123 Parma, Italy; 4 Orthopaedics Department,, AZ Alma Eeklo, Ringlaan 15, Eeklo, 9900 Eeklo, Belgium; 5 Colchester General Hospital, Turner Road, Essex, Colchester, CO4 5JL UK

**Keywords:** Isolated patellofemoral osteoarthritis, Patellofemoral arthroplasty, Total knee arthroplasty, Total knee replacement, Implant survival, Patient-reported outcome measures.

## Abstract

**Introduction:**

Isolated patellofemoral osteoarthritis (PFOA) remains a therapeutic challenge, with patellofemoral arthroplasty (PFA) and total knee arthroplasty (TKA) representing the main surgical options for end-stage disease. This systematic review applies the GRADE framework to evaluate comparative outcomes of PFA and TKA, providing evidence-based recommendations.

**Materials and methods:**

A PRISMA systematic search of Pubmed, Cochrane Library, and Google Scholar was conducted (2010–2025). RCTs, comparative cohort studies, and registry analyses reporting on PFA versus TKA for isolated PFOA were included. Primary outcomes were validated PROMs and implant survival at 2, 5, and 10 years. Secondary outcomes were complications, patient satisfaction, return to sport, and cost-effectiveness. Risk of bias was assessed with RoB 2 and ROBINS-I, and certainty of evidence using GRADE.

**Results:**

Ten studies were included (4 RCTs, 6 cohort studies; approximately 10,000 PFAs comprising registries). Moderate-certainty evidence indicated that PFA provides superior early PROMs, and short-term cost-effectiveness compared with TKA. PROMs converged between groups at mid- to long-term follow-up. Long-term data demonstrated a consistently higher revision risk for PFA with moderate certainty, with registry-based 10-year survival of 85% for PFA vs. 95% for TKA, continuing to worsen for PFA after 10 years. Complication rates were similar or lower after PFA, particularly for systemic medical events. Patient satisfaction and return to sport favored PFA short term but became comparable to TKA at mid-term.

**Conclusion:**

In carefully selected patients with isolated PFOA, modern onlay PFA yields faster recovery, superior early function, and short-term cost-effectiveness, supported by moderate-certainty evidence. These advantages are offset by a higher long-term revision risk compared with TKA, highlighting the need to inform patients of this trade-off. TKA remains the reference standard for patients with tibiofemoral disease or instability, supported by high-certainty evidence, and offers durable, predictable long-term outcomes in more heterogeneous patient populations.

**Level of evidence, II:**

Systematic GRADE (Grading of Recommendations, Assessment, Development and Evaluation) review of RCTs and observational studies.

**Supplementary Information:**

The online version contains supplementary material available at 10.1007/s00402-026-06217-3.

## Introduction

Isolated patellofemoral osteoarthritis (PFOA) is a degenerative knee condition that commonly causes anterior knee pain, stiffness, and functional limitation, particularly in middle-aged and older adults, with a higher prevalence among female patients [1]. It can be clinically evident even in its early or mild stages [2]. The radiographic prevalence of isolated PFOA is estimated to be about 25% in the general population aged > 20 years and 39% among people with knee symptoms aged > 30 years [3, 4]. Management strategies for isolated PFOA range from conservative approaches, such as physical therapy and pharmacologic interventions, to surgical options, including arthroscopic procedures, realignment surgeries, and prosthetic joint replacement [1, 5]. For patients with advanced, symptomatic disease who fail non-operative care and in whom joint preservation is no longer feasible, prosthetic solutions such as patellofemoral arthroplasty (PFA) [6,7] and total knee arthroplasty (TKA) [8] are considered.

Over the years, both TKA and PFA have evolved significantly through advancements in surgical techniques, implant design, and robotic assistance, along with a more refined understanding of patient selection and surgical indications [9–13]. These developments aim to improve surgical precision, enhance clinical outcomes, and implant survivorship. Despite these advancements, the optimal role of PFA versus TKA in the management of isolated PFOA remains debated, particularly in terms of long-term patient reported outcome measures (PROMs) and revision rates [9, 14, 15]. Decisions between PFA and TKA have profound implications for implant selection, patient outcomes, and risk profiles, making high-quality evidence synthesis essential for guiding orthopedic practice [9]. Several meta-analyses have compared PFA with TKA for isolated PFOA, but their conclusions are limited by heterogeneity in study design, patient selection, and reported outcomes [16–20]. While meta-analyses are valuable for quantifying outcomes, a GRADE (Grading of Recommendations, Assessment, Development and Evaluation) review contextualizes the evidence and translates it into practical guidance for clinical decision-making [5]. This systematic review synthesizes the available literature comparing PFA and TKA for isolated PFOA and applies the GRADE framework to assess the certainty of evidence, with the goal of providing transparent, evidence-based guidance. The objective was to generate clinically relevant recommendations that optimize patient selection and support surgical decision-making.

The primary objective was to compare PFA and TKA for isolated PFOA with respect to validated PROMs and implant survival at two, five, and ten years. Secondary objectives included the rates of complications, patient satisfaction, return to sport, and cost-effectiveness.

## Materials and methods

This systematic review followed a pre-registered, publicly accessible protocol (10.17605/OSF.IO/UQY2E).

### Search strategy

A systematic search of PubMed, Cochrane Library, and Google Scholar was conducted for studies published between January 2010 and September 2025 using keywords and MeSH terms for “patellofemoral arthroplasty”, “total knee arthroplasty”, and “patellofemoral osteoarthritis” (Supplementary Table 1). This timeframe was chosen to ensure the evidence reflects contemporary implant designs and surgical techniques. Manual reference checks of included studies were also performed. This review has followed a pre-specified PICO framework, summarizing the population, interventions, comparator, and outcomes of interest (Supplementary Table 2).

### Study selection and data collection

References were managed and formatted with a citation management software. Two reviewers independently screened titles, abstracts, and full texts against predefined eligibility criteria, with disagreements resolved by a third senior reviewer. Data were extracted on study characteristics, populations, interventions, follow-up, and outcomes.

### Outcomes of interest

Primary outcomes included validated PROMs and implant survival at 2, 5, and 10 years. Secondary outcomes included medical and surgical complications, patient satisfaction, return to sport, and cost-effectiveness.

### Data synthesis and statistical analysis

Evidence was synthesized narratively, separately for RCTs and observational studies. The certainty of evidence was assessed using the Grading of Recommendations Assessment, Development and Evaluation (GRADE) framework [21], providing transparent and clinically applicable recommendations. In accordance with GRADE guidance, RCTs are initially rated as high-certainty evidence and may be downgraded for limitations such as risk of bias, inconsistency, indirectness, imprecision, or publication bias. Observational studies are initially rated as low-certainty evidence but may be upgraded when they demonstrate a large effect size or a dose–response gradient. This approach allows structured comparison of evidence quality among study designs and outcomes. Risk of bias was evaluated with RoB 2 (Risk of Bias 2 tool) for RCTs and ROBINS-I (Risk Of Bias In Non-randomized Studies of Interventions) for observational studies. Risk of bias assessments were visualized using the robvis tool (available at https://mcguinlu.shinyapps.io/robvis/). Reporting adhered to PRISMA 2020 guidelines [22] and included a flow diagram as well as risk-of-bias summaries.

## Results

### Study selection

**Fig. 1:**
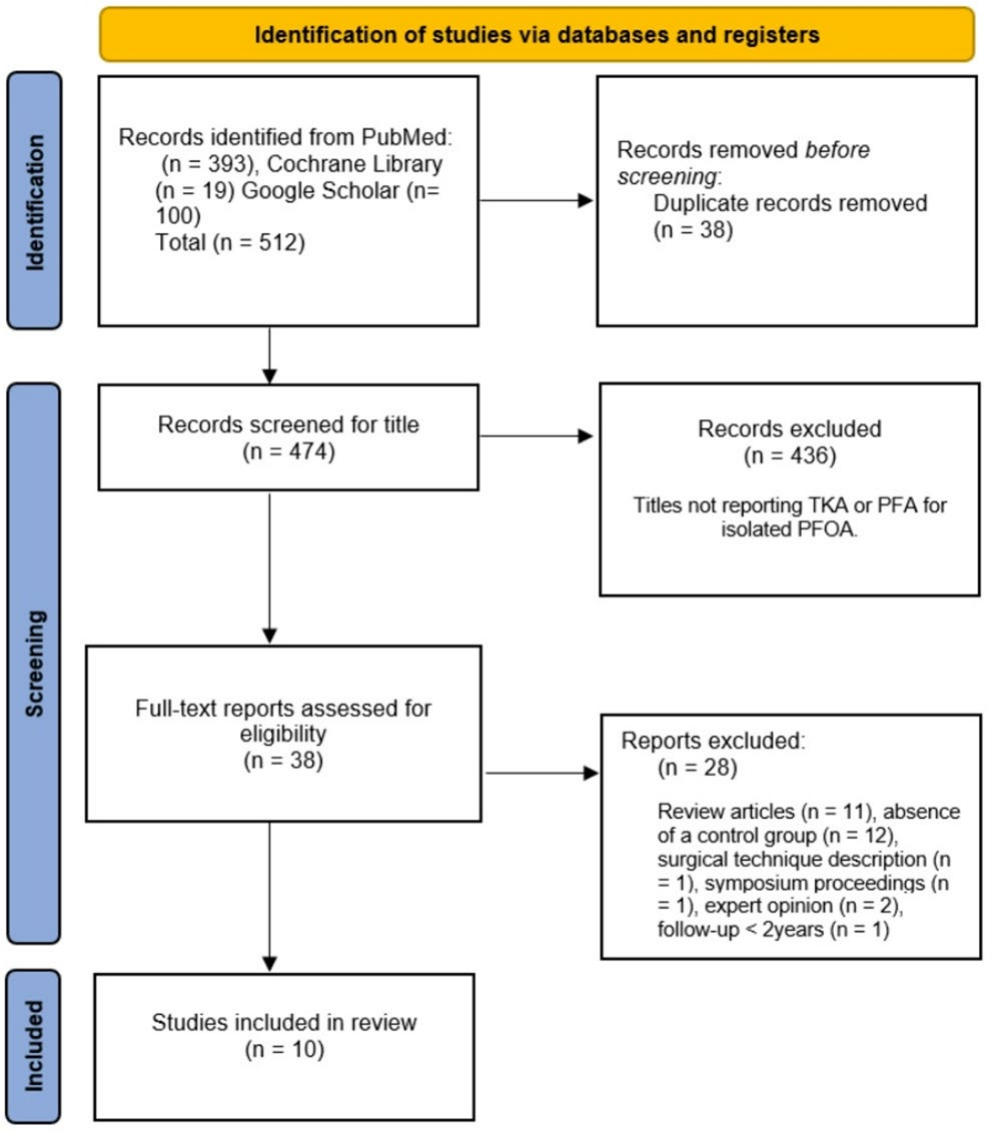
Displays the Flow Diagram illustrating the process of article selection following the PRISMA guidelines

A total of 512 potentially relevant citations were identified from three electronic databases (PubMed, *n* = 393; Cochrane Library, *n* = 19; Google Scholar, *n* = 100). After removal of 38 duplicates, 474 records remained. Screening of titles and abstracts excluded 453 citations, leaving 38 full-text articles for eligibility assessment. Of these, 28 were excluded for the following reasons: review articles (*n* = 11), absence of a control group (*n* = 12), surgical technique description (*n* = 1), symposium proceedings (*n* = 1), expert opinion (*n* = 2), and mean follow-up shorter than two years (*n* = 1). Ultimately, 10 studies [23–32] met the inclusion criteria. The study selection process is summarized in Fig. 1.

### Study characteristics (Table 1)

All ten included studies compared PFA with TKA for isolated PFOA (4 RCTs, 4 retrospective cohort studies, and 2 registry-based analyses). Overall, a total of 9,924 PFAs and 663,243 TKAs were included. The RCTs contributed 181 PFAs and 179 TKAs [23–26], retrospective cohort studies added 156 PFAs and 155 TKAs [27–30], and registries added 9,587 PFAs and 662,909 TKAs [31, 32]. While RCTs and cohort studies provided detailed clinical outcomes in relatively small, well-defined populations, the registries supplied large-scale data primarily focused on revision rates and complications. Observational studies were published between 2010 and 2022 [23–30]. Registry-based analyses were reported more recently in 2023 [31] and 2025 [32]. Only second-generation onlay PFA implants were used: Avon (Stryker), FPV (Wright), Gender Solutions PFJ (Zimmer). TKAs included a variety of designs, such as NexGen (Zimmer), Vanguard (Zimmer Biomet), Triathlon (Stryker), Medial Pivot (Wright), CR mobile (LINK), and Sigma PFC CR or PS (DePuy).

The mean patient age ranged from 50 years (SD ~ 5) in younger cohorts [29] to 72 years (SD ~ 9) in registry-based studies, with most RCTs reporting means of 64–65 years (SD ~ 8–9). Most studies reported a female predominance of 70–80%. Mean BMI ranged from 26.8 kg/m² [30] to 30 kg/m² [28], with most series reporting 27–29 kg/m² (SD ~ 4–5). Follow-up varied considerably: short term (1 year) [24], mid term (2–6 years) [23, 25, 26, 30, 31], and long term (9–15 years) [27, 32].

The primary outcomes analyzed among the studies included PROMs such as the Western Ontario and McMaster Universities Osteoarthritis Index (WOMAC), Oxford Knee Score (OKS), Knee injury and Osteoarthritis Outcome Score (KOOS), Short Form-36 Health Survey (SF-36), Forgotten Joint Score (FJS), quality-adjusted life years (QALY), cost-effectiveness analyses, and revision rates. Secondary outcomes included complications (infection, reoperation, thromboembolic events, and mortality), revision surgery, knee range of motion (ROM), radiographic loosening, patient satisfaction, and return to sport and activity, University of California Los Angeles (UCLA) activity score and Tegner activity scale.

**Table 1:** Study characteristics of comparative studies of patellofemoral arthroplasty versus total knee arthroplasty for isolated patellofemoral osteoarthritis.

Study (Year, Journal, Country)	Design	Intervention (PFA)	Control (TKA)	*N* (♀) PFA/TKA	Age, yrs PFA/TKA	BMI PFA/TKA	FU	Primary Outcome	Secondary Outcomes	Key Findings / Conclusion
*RCTs*										
Fredborg 2020, BJJ, Denmark [23]	RCT (cost-effectiveness)	Avon (Stryker)	PFC Sigma CR (DePuy)	50 (39) / 50 (38)	64.0 / 64.4	28.0 / 27.8	24 mo	Cost-effectiveness (QALY, EQ-5D)	SF-6D, reoperations	PFA had higher implant cost but lower overall cost, greater QALY gain at 12–24 mo, and shorter operative time (9.4 min, *p* = 0.005).
Joseph 2020, BJJ, UK [24]	RCT (pragmatic)	Avon, FPV, Zimmer	NexGen, Vanguard, Triathlon, Medial Pivot	31 (22) / 29 (26)	64.7 / 64.4	28.9 / 29.2	60 mo	WOMAC (12 mo)	OKS, EQ-5D, AKSS, UCLA, survival	Similar functional outcomes at 12, 24 and 60 mo; no superiority of PFA.
Odgaard 2018, CORR, Denmark [25]	RCT	Avon (Stryker)	PFC Sigma CR (DePuy)	50 / 50 (77% ♀)	64	28 / 28	24 mo	SF-36 (pain)	KOOS, OKS, ROM, revisions	PFA showed early advantages in PROMs and ROM; at 2 yrs, only KOOS remained superior.
Odgaard 2022, CORR, Denmark [26]	RCT	Avon (Stryker)	PFC Sigma CR (DePuy)	50 (39) / 50 (38)	64 / 65	28.0 / 27.8	6 yrs	SF-36 (pain)	KOOS, OKS, ROM, revisions	Early PROM advantages for PFA persisted over 6 yrs (AUC analysis); at 6 yrs, outcomes were similar except vitality remained higher for PFA.
*Cohorts*										
Clement 2019, BJJ, UK [27]	Retrospective, propensity-matched	Avon (Stryker)	Triathlon (Stryker)	54 (49) / 54 (46)	62.4 / 64.0	NR	9.2 yrs (8–15)	OKS	SF-12, satisfaction, survival	Function and satisfaction were comparable; PFA had shorter hospital stay but a nonsignificant trend toward higher revision risk.
Kamikovski 2019, JOA, Canada [29]	Retrospective, case-matched	Avon, Gender Solution PFJ	NR	23 (3) / 23 (4)	50.4 / 50.5	28.5 / 28.2	5.3 yrs	KOOS, WOMAC (< 55 yrs)	UCLA, Tegner	TKA showed superior outcomes at 1 year, but PFA achieved comparable results by 2 yrs, considered a bone-preserving option in patients < 55 yrs.
Lin 2021, Orthop Surg, China [30]	Retrospective, case-matched	PFJ (Zimmer)	CR mobile (LINK)	56 (40) / 56 (41)	59.2 / 58.6	26.8 / 27.0	1 and 3 yrs	FJS, KSS, ROM	Radiographic loosening	PFA yielded higher ROM, FJS, and KSS at 1–3 yrs; benefits were most evident in older patients with lower BMI.
Dahm 2010, Am J Orthop, USA [28]	Retrospective cohort	Avon (Stryker)	Zimmer, Sigma CR/PS (DePuy)	23 / 22	60 / 69	30	27–29 mo	KSS, UCLA, Tegner	Blood loss, LOS, satisfaction	Outcomes were comparable; PFA had less blood loss, shorter hospital stay, higher UCLA scores, and no revisions.
*Registries*										
Serino 2023, The Knee, USA [31]	Registry (PearlDiver matched)	PFA	TKA	1,768 / 1,768	57.5 / 57.6	NR	5 yrs	Cost	Complications, revisions	PFA had higher 5-yr revision risk (9.9% vs. 4.2% for TKA) but fewer ED visits and lower pneumonia rates; other medical complications were comparable.
Vella-Baldacchino 2025, BJJ, UK [32]	NJR registry, weighted analysis	PFA	TKA	7,819 / 662,141	Younger / Older	NR	≤ 10 yrs	30-day complications	Survival, revisions	PFA was safer perioperatively, with lower risks of infection, DVT/PE, and death, but had higher 10-yr revision risk (85% vs. 95% survival for TKA). Best considered in selected patients.

*PFA* patellofemoral arthroplasty, *TKA* total knee arthroplasty, *RCT* randomized controlled trial, *BJJ* bone and joint journal, *CORR* clinical orthopaedics and related research, *JOA* journal of arthroplasty, *Am J Orthop* American journal of orthopedics, *NJR* National joint registry, *CR* cruciate-retaining, *PS* posterior-stabilized, *N* number of patients, ♀ female; yrs years, *FU* follow-up, *QALY* quality-adjusted life year, *EQ-5D* EuroQol 5-dimension, *SF-6D* Short Form 6-dimension, *SF-36* Short Form 36, *KOOS* Knee Injury and Osteoarthritis Outcome Score, *OKS* Oxford Knee Score, *WOMAC* Western Ontario and McMaster Universities Osteoarthritis Index, *AKSS* American Knee Society Score, *KSS* Knee Society Score, *UCLA* University of California Los Angeles activity scale, *FJS* Forgotten Joint Score, *ROM* range of motion, *AUC* area under the curve, *LOS* length of stay, *ED* emergency department, *DVT* deep vein thrombosis, *PE* pulmonary embolism, *NR* not reported

### PROMs and patient satisfaction (Table 2)

PFA was associated with earlier improvements in pain, function, and joint-specific quality of life, although satisfaction and PROMs tended to converge with TKA at longer follow-up. Among RCTs, PFA demonstrated better early PROMs than TKA, particularly in SF-36 bodily pain, KOOS symptoms, OKS, and range of motion (ROM) recovery during the first postoperative year [23, 25]. These benefits were sustained in time-weighted analyses up to six years, although at individual 2- and 6-year follow-ups, differences narrowed and only selected outcomes (e.g., KOOS symptoms, SF-36 vitality) remained superior [26]. The pragmatic PAT RCT [24] reported no significant differences in WOMAC, OKS, or EQ-5D scores up to 5 years, highlighting inconsistency among RCT findings [24, 26]. Observational cohorts generally supported functional advantages of PFA, with higher FJS, KSS, and UCLA activity levels, particularly in younger and more active patients [28–30]. However, long-term matched analyses (mean 9.2 years of follow-up) showed no differences in PROMs or patient satisfaction between groups [27]. Registry-based studies did not report PROMs. Patient satisfaction generally paralleled PROMs, with early advantages for PFA but convergence with TKA over time; long-term follow-up showed comparable satisfaction rates [27].

**Table 2:** Patient-Reported outcome measures (PROMs) and patient satisfaction

Study (Year, Journal, Country)	Design / FU	PROMs (PFA vs. TKA)	Satisfaction
*RCTs*			
Odgaard 2018, CORR, Denmark [25]	RCT, 2 yrs	PFA better early PROMs (SF-36 pain, OKS, KOOS symptoms) and faster ROM recovery ≤ 9 mo; at 2 yrs only KOOS symptoms superior. PFA regained pre-op ROM; TKA lost ~ 10°.	Not directly measured; PROMs suggest better early satisfaction with PFA, convergence by 2 yrs.
Odgaard 2022, CORR, Denmark [26]	RCT, 6 yrs	Time-weighted AUC (0–6 yrs): PFA better (SF-36 pain, OKS, KOOS symptoms). At isolated 6 yrs: only SF-36 vitality superior for PFA.	Not directly measured; interpretation: higher QoL over 6 yrs, groups similar at 6 yrs.
Joseph 2020, BJJ, UK [24]	RCT (pragmatic), 5 yrs	No significant differences (WOMAC, OKS, EQ-5D) at 12, 24, or 60 mo.	Similar overall; at 60 mo, more TKA patients are “very satisfied” (60% vs. 22%), NS.
Fredborg 2020, BJJ, Denmark [23]	RCT (cost-effectiveness), 2 yrs	EQ-5D favored PFA at 1–2 yrs, with clinically relevant gains; SF-6D also favored PFA (more at 12 mo than 24 mo).	Not directly measured; higher QALYs imply greater satisfaction with PFA.
*Cohorts*			
Clement 2019, BJJ, UK [27]	Matched cohort, 9.2 yrs	Equivalent OKS and SF-12 at 1 year and final FU; no long-term PROMs difference.	Satisfaction similar (87% PFA vs. 78% TKA), NS.
Dahm 2010, Am J Orthop, USA [28]	Retrospective, 27–29 mo	Higher Tegner and UCLA scores with PFA; no difference in KSS, pain, or ROM.	No difference in satisfaction (*p* = 0.66).
Kamikovski 2019, JOA, Canada [29]	Retrospective, 2 yrs	TKA superior at 1 year (KOOS, WOMAC); at 2 yrs PFA caught up, showing greater improvement 1–2 yrs.	Not directly measured; conclusion: PFA valid in younger patients, comparable at 2 yrs.
Lin 2021, Orthop Surg, China [30]	Retrospective, 3 yrs	PFA superior in FJS, KSS, and ROM at 1 and 3 yrs; predictors: older age = ↑FJS, higher BMI = ↓FJS.	No direct measure, but higher FJS suggests better satisfaction with PFA.
*Registries*			
Serino 2023, Knee, USA [31]	Registry, 5 yrs	not reported.	not reported.
Vella-Baldacchino 2025, BJJ, UK [32]	NJR registry, ≤ 10 yrs	not reported.	not reported.

*FU* follow-up,* yrs* years,* mo* months,* PFA* patellofemoral arthroplasty,* TKA* total knee arthroplasty,* RCT* randomized controlled trial,* BJJ* Bone & Joint Journal, CORR Clinical Orthopaedics and Related Research, JOA Journal of Arthroplasty, Am J Orthop American Journal of Orthopedics, PROMs Patient-Reported Outcome Measures, OKS Oxford Knee Score, KOOS Knee injury and Osteoarthritis Outcome Score, WOMAC Western Ontario and McMaster Universities Osteoarthritis Index, KSS Knee Society Score, FJS Forgotten Joint Score, SF-36 Short Form–36 Health Survey, SF-12 Short Form–12 Health Survey, EQ-5D EuroQol 5-Dimensions questionnaire, SF-6D Short Form–6 Dimensions, SG Standard Gamble, VAS Visual Analogue Scale, QALY Quality-Adjusted Life Year, MCID Minimal Clinically Important Difference, UCLA University of California Los Angeles Activity Scale, ROM Range of Motion, NS non significant.

### Implant survival (Table 3)

Evidence consistently shows higher revision rates after PFA, mainly due to progression of tibiofemoral osteoarthritis, unexplained pain, or aseptic loosening. RCTs suggest PFA achieves excellent short-term survivorship, with revision-free survival comparable to TKA at two years [24]. In Odgaard’s 2018 trial [25], survival was 96% for PFA versus 100% for TKA, while six-year follow-up [26] showed a persistent but nonsignificant difference (90% vs. 96%). The pragmatic PAT RCT [24] reported no revisions at five years, and a cost-effectiveness trial recorded very few events, limiting survival analysis [23]. Collectively, these trials indicate PFA may carry a modestly higher mid-term revision risk, though RCTs were underpowered for survival endpoints and true differences remain uncertain. Observational cohorts confirmed excellent short-term survivorship [28–30], with no revisions up to 3 years (100% survival for both PFA and TKA). Longer-term matched analyses [27] (mean follow-up 9.2 years) showed higher revision rates in PFA (92.3% vs. 100% at 10 years; *p* = 0.10), despite similar functional outcomes, suggesting revision risk becomes evident with longer follow-up. Registry studies reported 5-year survival of 90.1% for PFA and 95.8% for TKA [31], while the UK NJR–HES linkage study [32] reported 10-year survival of 85% and 95%, respectively.

**Table 3:** Implants survival at two, five and ten years

Study (Year, Journal, Country)	Design / FU	2-year survival	5-year survival	10-year survival	Notes
*RCTs*					
Odgaard 2018, CORR, Denmark [25]	RCT, 2 yrs	PFA 96% (2/50 revisions); TKA 100% (1 fracture, no revision)	–	–	No direct survivorship analysis; survival estimated from complications.
Odgaard 2022, CORR, Denmark [26]	RCT, 6 yrs	PFA 96% (2/50 revisions); TKA 100%	PFA 90% (5 revisions); TKA 96% (2 revisions)	–	Revision risk higher for PFA, not statistically significant.
Joseph 2020, BJJ, UK [24]	RCT (pragmatic), 5 yrs	PFA 100%; TKA 100%	PFA 100%; TKA 100%	–	No revisions at 12, 24, or 60 mo.
Fredborg 2020, BJJ, Denmark [23]	RCT (cost-effectiveness), 2 yrs	1 PFA revision; TKA no revisions	–	–	Survival rates not explicitly reported.
*Cohorts*					
Clement 2019, BJJ, UK [27]	Matched cohort, 9.2 yrs	–	PFA 94.2%; TKA 100%	PFA 92.3%; TKA 100%	Difference not statistically significant (*p* = 0.10).
Dahm 2010, Am J Orthop, USA [28]	Retrospective, 27–29 mo	PFA 100%; TKA 100%	–	–	No revisions in either group.
Kamikovski 2019, JOA, Canada [29]	Case-matched, 2 yrs	–	–	–	No revisions, but sample (23 vs. 23) underpowered for survival analysis.
Lin 2021, Orthop Surg, China [30]	Case-matched, 3 yrs	PFA 100%; TKA 100%	–	–	No revisions or reoperations.
*Registries*					
Serino 2023, Knee, USA [31]	Registry (PearlDiver), 5 yrs	–	PFA 90.1%; TKA 95.8% (Kaplan-Meier)	–	PFA is associated with significantly higher 5-year revision risk.
Vella-Baldacchino 2025, BJJ, UK [32]	NJR registry, ≤ 10 yrs	PFA 94%; TKA 98%	PFA 90%; TKA 96%	PFA 85%; TKA 95%	PFA is less durable long term. Early revisions for pain, later failures from tibiofemoral OA progression. Survival improving with newer implants/selection.

### Complications (Table 4)

PFA has a favorable short-term safety profile and perioperative advantages, but long-term durability is inferior to TKA. Short-term complication rates (within 2–3 years) were low and similar between groups, with no consistent differences in surgical or medical adverse events. Large registry data from the UK NJR [32] suggest that PFA is associated with fewer systemic complications and lower 30-day mortality compared with TKA. Infection rates were comparable across studies, while periprosthetic fracture was rare and reported only sporadically for both procedures. The key difference is revision risk, which is consistently higher after PFA beyond 5 to 10 years [27, 32].

**Table 4:** Complication rates

Study (Year, Journal, Country)	Design / FU	PFA – Complications	TKA – Complications	Notes
*RCTs*				
Fredborg 2020, BJJ, Denmark [23]	RCT, 2 yrs	1 revision, 2 reoperations (no fractures)	0 revisions, 4 reoperations (2 MUA, 1 fracture)	Low event rate; no group difference.
Joseph 2020, BJJ, UK [24]	RCT (pragmatic), 5 yrs	4 superficial infections; no reoperations	5 superficial infections; 4 interventions (arthroscopy, MUA, aspiration/steroid)	Comparable complication rate.
Odgaard 2018, CORR, Denmark [25]	RCT, 2 yrs	2 revisions (1 PFA exchange, 1 TKA conversion); 2 patellar reoperations; 2 unrelated deaths	0 revisions; 5 reoperations (3 MUA, 1 arthroscopy, 1 fracture)	Similar complication rate.
Odgaard 2022, CORR, Denmark [26]	RCT, 6 yrs	5 revisions (10%); 5 reoperations	2 revisions (4%); 6 reoperations	No significant difference in revision/reoperation risk at 6 yrs.
*Cohorts*				
Clement 2019, BJJ, UK [27]	Retrospective, 9.2 yrs	5 revisions at 6 yrs (OA progression, pain, fracture); 3 reoperations	1 revision at 11 yrs (tibial loosening); survival 100% at 10 yrs	Higher PFA revision, not statistically significant.
Dahm 2010, Am J Orthop, USA [28]	Retrospective, 27–29 mo	No complications/revisions	1 DVT (4%), 1 MUA (4%)	PFA = less blood loss, shorter stay, no revisions.
Kamikovski 2019, JOA, Canada [29]	Retrospective, 2 yrs	No revisions	No major complications	Both procedures safe, no differences.
Lin 2021, Orthop Surg, China [30]	Retrospective, 3 yrs	No complications/revisions	No complications/revisions	Both safe; PFA had higher ROM and FJS.
*Registries*				
Serino 2023, The Knee, USA [31]	PearlDiver database, 5 yrs	Revision 9.9% at 5 yrs; ↓ pneumonia, ↓ ED visits	Revision 4.2% at 5 yrs	PFA cheaper overall but ↑ revision risk.
Vella-Baldacchino 2025, BJJ, UK [32]	NJR + HES, ≤ 10 yrs	↓ 30-day complications (RTI, DVT/PE, UTI, wound infection, death); ↑ stroke risk; higher revision risk (15% at 10 yrs)	↑ early systemic complications; lower revision risk (5% at 10 yrs)	PFA safer perioperatively; TKA more durable long term.

*FU* follow-up,* yrs* years, mo months,* PFA* patellofemoral arthroplasty,* TKA* total knee arthroplasty,* RCT* randomized controlled trial,* BJJ* Bone & Joint Journal,* CORR* Clinical Orthopaedics and Related Research,* JOA* Journal of Arthroplasty,* Am J Orthop* American Journal of Orthopedics,* ROM *range of motion,* FJS* forgotten joint score,* NJR* National Joint Registry,* HES* Hospital Episode Statistics,* ED* emergency department,* DVT* deep vein thrombosis,* PE* pulmonary embolism,* UTI* urinary tract infection,* RTI* respiratory tract infection,* MUA* manipulation under anesthesia

### Return to sport

PFA was associated with earlier return to sport and higher activity levels, reflected in superior UCLA and Tegner scores at short-term follow-up, particularly in younger patients [28]. These advantages diminished with time, as the pragmatic PAT trial [24] reported comparable activity levels between PFA and TKA at 5 years. Most patients resumed recreational activity after PFA, with the most commonly reported postoperative sporting activities in both groups were walking, cycling, and swimming [28].

### Cost-effectiveness

Economic evidence suggests that PFA may be cost-effective in the short term for appropriately selected patients. The Danish cost-effectiveness RCT reported lower overall costs, shorter operative time, and greater QALY gains with PFA at one year despite higher implant costs [23]. In contrast, the U.S. registry analysis found PFA associated with lower short-term healthcare utilization but higher 5-year revision rates [31]. Implant survival remains the key determinant of long-term economic value, with revision risk limiting sustained cost-effectiveness.

### Indications and patient selection

Indications for PFA were consistently restricted to isolated patellofemoral osteoarthritis with preserved tibiofemoral compartments. None of the studies included patellofemoral instability as an indication. Dahm et al. [28] reported that many patients presented with trochlear dysplasia and patella alta, features often associated with instability, but in this cohort they were observed only in the setting of isolated PFOA and were not considered inclusion criteria. In their cohort, the mean tibiofemoral Kellgren–Lawrence (KL) score was 1; patients were included only if KL ≤ 2. Odgaard et al. [25, 26] excluded patients with KL grade 3–4 tibiofemoral changes and those with tibiofemoral full-thickness cartilage lesions > 6 mm. Other studies applied radiographic or intraoperative exclusion criteria. Joseph et al. [24] excluded tibiofemoral OA found intraoperatively, while Lin et al. [30] excluded “major tibiofemoral OA” radiographically. Fredborg et al. [23] and Kamikovski et al. [29] required radiographic confirmation of isolated patellofemoral disease without tibiofemoral involvement, but did not specify radiographic criteria.

### Risk of bias assessment (Supplementary tables 3–4, Figs. 1–2)

Overall, the four RCTs demonstrated low to moderate risk of bias [23–26]. Odgaard et al. 2018 [25] and Fredborg et al. [23] were judged at low risk of bias in all domains, maintained high follow-up with robust time-weighted analyses; whereas Joseph et al. [24] and Odgaard et al. 2022 [26] had “some concerns” related to blinding and survival endpoints were underpowered (Supplementary Tables 3 and Fig. 1). Observational studies showed non-randomized allocation, residual confounding, and absence of blinding. Clement et al. [27] and Kamikovski et al. [29], were rated as moderate risk, while Lin et al. [30] and Dahm et al. [28] were judged to be at serious risk in domains such as confounding and patient selection. Registry-based studies [31, 32] were assessed at moderate risk of bias, given their reliance on administrative coding and potential residual confounding, though large sample sizes and near-complete follow-up strengthened confidence in revision rates and complications (Supplementary Tables 4 and Fig. 2).

### Certainty of evidence according to specific outcomes (Table 5)

Isolated PFAO represents the primary indication for PFA, ideally in patients with bone-on-bone patellofemoral disease, preserved tibiofemoral compartments, and neutral alignment. However, the precise thresholds of acceptable varus/valgus deformity remain uncertain, and the limits of coronal knee deformity that still permit successful PFA are not well defined. Evidence certainty is moderate for PFA in these selected patients. TKA carries high-certainty evidence, supported by durable outcomes and broader indications, and remains the reference standard when tibiofemoral osteoarthritis, malalignment, or patellofemoral instability are present. The certainty of evidence (GRADE) varied by outcome. PROMs and QoL were graded as moderate certainty, supported by consistent early advantages for PFA in two RCTs [25, 26], but downgraded for inconsistency, as the pragmatic PAT trial [24] showed no advantage of PFA over TKA. Revision risk was graded as moderate certainty; RCTs were underpowered for rare events given small sample sizes [23–26], while registry studies consistently showed higher revision risk for PFA but were downgraded for potential confounding and indirectness [31, 32]. Short-term medical complications and length of stay were graded as moderate certainty. Registry and cohort data indicated no increased complication risk and possible perioperative advantages with PFA. Knee ROM was graded as moderate certainty. PFA demonstrated superior short-term ROM, but this advantage diminished over time; certainty was downgraded from high because of inconsistency across RCTs and imprecision from small sample sizes. Return to sport was graded as low to moderate certainty, based on small cohorts and underpowered secondary outcomes in RCTs. Cost-effectiveness was graded as moderate certainty, supported by RCT-based analyses and registry modeling, though downgraded for indirectness and limited generalizability across healthcare systems [23, 31].

**Table 5:** Risk of bias and certainty of evidence according to specific outcomes

Outcome	What the studies show	Certainty (GRADE)	Notes
1. Patient selection — ideal candidates	PFA: best for isolated, bone-on-bone patellofemoral OA, preserved tibiofemoral joint, neutral alignment, stable joints. Predictors: ↑age → ↑FJS; ↑BMI → ↓FJS. TKA: preferred if tibiofemoral OA, malalignment, instability, inflammatory arthritis, high OA progression risk.	↑ ↓ Moderate (PFA) ↑ ↓ High (TKA)	TKA remains the established standard, supported by long-term data.
2. Functional outcomes	Early (≤ 2 yrs): PFA superior [25,28,30]. Time-weighted: sustained PFA advantage up to 6 yrs [26]. PAT RCT: no difference at 1–5 yrs [24]. Mid-term (≥ 3–6 yrs): convergence; only SF-36 vitality favored PFA at 6 yrs [26].	↑ ↓ Moderate	Started high (RCTs) → downgraded for inconsistency, Odgaard [25,26] vs. PAT [24].
3. Implant survival (mid- to long-term)	RCT: no significant difference at 6 yrs (PFA 10% vs. TKA 4%, *p* = 0.24) [26]. Registries : consistently higher PFA revisions (≈ 10% vs. 4% at 5 yrs; 85% vs. 95% at 10 yrs) [31;32]. Matched cohort: trend ↑ PFA revisions at 9 yrs 27[].	↑ ↓ Moderate	Consistent signal, but RCTs underpowered (imprecision) and registries subject to residual confounding. Overall: moderate certainty PFA = ↑mid- to long-term revision risk.
4. Complications	Medical: PFA = equal or lower systemic events (↓RTI, DVT/PE, UTI, wound infection, mortality) [31,32]. Surgical: RCTs and cohorts show no excess early surgical complications with PFA.	↑ ↓ Moderate	Downgraded for registry confounding and low event rates in RCTs, but signal consistent.
5. Return to sport / activity	PFA: higher activity scores (UCLA/Tegner) [28] and earlier return to sport at 1–2 yrs [25]. Differences diminish by 3–6 yrs.	↑ ↓ Low–Moderate	Reliance on small cohorts and underpowered secondary outcomes in RCTs.
6. Cost / cost-effectiveness	RCT: short-term (1–2 yrs), PFA dominant (↓cost, ↑QALYs) [23]. Registries: PFA cheaper despite higher revisions [31].	↑ ↓ Moderate	Generalisability across health systems uncertain. Long-term evidence modeled, not trial-based.
7. Causes of PFA failure	Common: tibiofemoral OA progression, persistent pain, maltracking/instability, aseptic loosening.	↑ ↓ Moderate	Evidence is consistent among cohort and registry studies, though mainly observational. Long-term survivorship is limited primarily by progression of tibiofemoral OA rather than certain implant-related factors.

### Summary GRADE practice recommendations for PFA versus TKA in isolated PFOA

PFA: Conditional recommendation in carefully selected patients with isolated PFOA, particularly when the goal is to maximize early function, range of motion, and short-term cost-effectiveness. Certainty: moderate (supported by RCTs and cohort studies; consistently higher long-term revision risk demonstrated in registries).

TKA: Strong recommendation when tibiofemoral osteoarthritis, malalignment, instability, or other contraindications to PFA are present. Certainty: high (robust, consistent outcomes and superior long-term survivorship among broader patient populations).

## Discussion

### GRADE assessment (Table 5)

This systematic review applied the GRADE framework to compare PFA and TKA in isolated PFOA. For each clinically relevant outcome, we evaluated both the direction and magnitude of effect and the certainty of the underlying evidence (high, moderate, low, or very low) by systematically assessing risk of bias, inconsistency, indirectness, imprecision, and publication bias. We included four RCTs [23–26] and six comparative cohort/registry-based studies [27–32]. With moderate certainty, PFA was associated with superior early PROMs. These benefits were downgraded for inconsistency, given that the Odgaard trials [25, 26] demonstrated clear early and time-weighted advantages to six years for PFA compared to TKA, whereas the pragmatic PAT trial [24] found no difference at one to five years. Implant survival carried moderate certainty for less favorable outcomes for PFA compared to TKA, but RCTs were underpowered to detect significant differences in revision rates, while large registry studies consistently demonstrated higher mid- to long-term revision risks for PFA (90% vs. 96% at 5 years; 85% vs. 95% at 10 years, for PFA and TKA respectively) [31, 32]. Certainty was downgraded for indirectness related to registry confounding and limited generalizability among health systems [33]. Complication risk were graded as moderate certainty. Medical complications were similar or lower with PFA in registry analyses [31, 32], while surgical complication rates were equivalent among RCTs and observational studies [23–30]. Return-to-sport outcomes favored PFA in the short term, but with low to moderate certainty given reliance on small cohorts and underpowered secondary outcomes in RCTs.

### Patient-reported outcomes (PROMs) and satisfaction (Table 2)

Among RCTs, PFA has demonstrated faster early functional recovery and QoL improvement. Odgaard et al. [25, 26] reported superior SF-36, KOOS, and OKS scores with PFA, sustained in time-weighted analyses to six years [26]. In contrast, the pragmatic PAT RCT, using multiple implant designs in routine practice, found no significant differences at one to five years [24]. Observational cohorts [29, 30] reported higher FJS and KSS with PFA in the first three years, particularly in younger patients. Overall, moderate-certainty evidence indicates that PFA provides superior early gains, with outcomes converging with TKA over time [27]. The short-term effectiveness of second-generation PFA implants has been confirmed in comparative studies [19, 20], showing superior functional recovery and higher activity levels limited to the first two years. Recent single-implant series with lower-level evidence employing third-generation onlay designs have demonstrated excellent early PROMs following PFA [34, 35]. Patient satisfaction generally paralleled PROMs, with early advantages for PFA but convergence with TKA over time; long-term follow-up showed comparable satisfaction rates [27].

### Implant survival **(**Table 3)

Short-term survival is excellent for both procedures. In the Odgaard RCT [26], revision-free survival at 6 years was 90% for PFA compared with 96% for TKA with a non-significant difference, while in the PAT trial [24] both groups achieved 100% survival at 5 years. Observational cohorts similarly demonstrated near 100% survival at 2 to 3 years [28–30]. Mid- to long-term registry data reported 5-year survival of 90% for PFA and 96% for TKA (*p* = 0.003) in the U.S [31]. , and a 10-year survival of 85% for PFA and 95% for TKA in the UK NJR [32]. The Norwegian Arthroplasty Register confirms inferior long-term survivorship of PFA, declining to 73% for PFA versus 92% for TKA after 15 years of follow-up, but these results reflect procedures performed for knee osteoarthritis in general rather than exclusively for isolated PFOA in Norway [33]. The most frequent cause of PFA failure was progression of tibiofemoral osteoarthritis, accounting for nearly half of PFA revisions. A recent population-based NJR/HES cohort study demonstrated that surgeons performing > 5 PFAs annually had significantly lower revision rates, highlighting the importance of surgical volume in optimizing PFA outcomes [36]. Overall, modern PFA onlay implants demonstrate acceptable survivorship [37, 38]. The survival data confirm PFA durability in the short- to mid-term but consistently inferior long-term survivorship compared with TKA. Meta-analyses including RCTs underpowered for survivorship outcomes and low-quality observational studies have shown no significant difference in revision rates between PFA and TKA for isolated PFOA in the short- to mid-term with second-generation implants, although revision events were rare in both groups within the pooled analyses [16, 19].

### Complication rates (Table 4)

RCTs demonstrated no increase in surgical complication rates with PFA compared to TKA [24–26]. Cohorts reported lower perioperative morbidity, including less blood loss and shorter length of stay [28, 30], findings confirmed in a recent meta-analysis [20]. Registry analyses confirmed this trend also for medical complications, the UK NJR–HES linkage [32] showed reduced 30-day risks of respiratory infection, venous thromboembolism, wound infection, and mortality with PFA relative to TKA. Thus, evidence of moderate certainty indicates that PFA is at least as safe perioperatively as TKA and may carry fewer systemic risks. Robotic-assisted PFA has been proposed to improve accuracy and reduce complications compared with manual techniques. A recent comparative study reported that manual PFA was associated with more 90-day complications and longer hospital stays, while revision rates remained similar between robotic and manual approaches. Notably, the overall PFA revision rate exceeded Michigan Arthroplasty Registry Collaborative Quality Initiative benchmarks for unicompartmental and total knee arthroplasty [39]. A recent analysis of the Australian Orthopaedic Association National Joint Replacement Registry also found that robotic assistance for PFA did not improve early revision rates when compared to manual PFA [40].

### Return to sport and activity

Evidence suggests that PFA facilitates an earlier return to sport activity compared with TKA particularly among younger patients, with low-moderate evidence [28]. These advantages appear to diminish over time, with the PAT trail [24] showing comparable activity levels at five years. Recent systematic reviews demonstrated that most patients are able to resume recreational activity after PFA, particularly low-impact sports [41, 42]. Approximately 58.6% of patients resumed sport within six months after PFA, and among those who returned, 74.8% achieved or surpassed their preoperative activity level [41]. Future high-quality studies with standardized definitions of return-to-sport are needed to clarify the long-term influence of activity on implant survival.

### Cost-Effectiveness

Economic evidence suggests that PFA may be cost-effective in the short term for appropriately selected patients compared to TKA. The Danish RCT showed lower cost and better outcomes at one year [23], while a U.S. registry analysis found PFA less expensive but with higher 5-year revision risk [31]. Certainty was graded moderate, as evidence depends on modeling and may not generalize among health systems. A U.S. model indicated cost-effectiveness in younger patients and greater lifetime QALYs with PFA if revision rates decline and QoL improvements are sustained [43]. Implant survivorship remains the key determinant of long-term economic value of PFA for isolated PFOA.

### Patient selection

Careful patient selection remains the cornerstone of successful PFA. PROM improvements are most pronounced in patients younger than 55 years [29], although registry data suggest younger patients remain more likely to fail because of tibiofemoral OA progression [31, 33]. Lin et al. [30] reported that older age predicted higher FJS, whereas higher BMI predicted lower scores, and registry cohorts confirm obesity as a risk factor for revision [32]. The ideal candidate is therefore a non-obese patient, with isolated PFOA, preserved tibiofemoral compartments, neutral alignment, and no patellofemoral instability [44]. Marullo et al. [45] observed that outcomes were paradoxically best at the extremes of age, patients < 55 years demonstrated superior clinical results, minimal OA progression, and high survivorship, whereas those > 76 years achieved excellent implant survival and satisfactory function. By contrast, patients aged 56 to 65 years carried the highest risk of revision. Thus, PFA may be particularly effective in carefully selected younger and older patients. Historically, contraindications to PFA included uncorrected patellofemoral instability or malalignment, patella baja, fixed limitation of knee ROM (minimum of 10° extension deficit to 110° flexion), uncorrected tibiofemoral malalignment (valgus > 8° or varus > 5°), and tibiofemoral arthritis beyond KL grade 1 [46].

### Future research

Certain evidence gaps remain. Adequately powered RCTs with long-term follow-up are needed to assess the survival of second and third-generation onlay PFA against modern TKA implants. Design-specific outcomes of PFA should be evaluated through large registry analyses with implant-specific data. Predictive models for tibiofemoral osteoarthritis progression after PFA should be studied integrating demographic, radiographic, and biomechanical risk factors. A matched cohort study has reported lower patient satisfaction after TKA following PFA compared with primary TKA, and higher-level evidence is needed to confirm this finding [47].

### Limitations

This review has certain limitations. We acknowledge that no meta-analysis was performed due to the clinical and methodological heterogeneity of included studies (Supplementary Materials Table 5). The studies differed in design (RCTs, retrospective cohorts, and large registries), indications and patient selection criteria, implant designs, follow‑up duration, and primary outcomes. Whereas previous meta-analyses have predominantly pooled a limited number of outcomes to generate summary effect estimates, our GRADE-based review was designed to provide a more comprehensive and decision-oriented synthesis. By assessing PROMs, implant survivorship, complications, return to sport, and cost-effectiveness within a unified GRADE framework, we not only described the direction and magnitude of effects but also rated the certainty of evidence for each outcome (high, moderate, low, or very low). This outcome-specific grading offers clinicians a more comprehensive, structured basis, for interpreting the current literature and individualising treatment decisions in patients with isolated PFOA. The number of RCTs was small and underpowered for survivorship endpoints; however, we included large registry datasets that provide robust estimates of revision risk. Follow-up duration varied and long-term outcomes beyond ten years were limited; nonetheless, the review integrates the most recent registry evidence extending after 15 years, which offers valuable insight into implant survival. Outcome reporting among studies was heterogeneous, particularly for PROMs, but multiple high-quality RCTs contributed validated PROMs that allow meaningful comparisons. Cost-effectiveness findings may be influenced by health system heterogeneity, yet the inclusion of both trial-based [23] and registry-based economic analyses from different healthcare systems [31] enhances their relevance.

## Conclusion

In carefully selected patients with isolated PFOA, modern onlay PFA provides short-term advantages in functional recovery and cost-effectiveness, with favorable perioperative safety, supported by moderate-certainty evidence. However, PFA carries a higher risk of mid- to long-term revision compared with TKA. These benefits and risks should be discussed with patients. TKA remains the reference standard for patients with tibiofemoral disease or patellofemoral instability, supported by high-certainty evidence, and offers durable, predictable long-term outcomes in more heterogeneous patient populations.

## Supplementary Information

Below is the link to the electronic supplementary material.

Supplementary Material 1

## Data Availability

No datasets were generated or analysed during the current study.

## References

[1] Donell ST, Glasgow MM (2007) Isolated patellofemoral osteoarthritis. Knee 14(3):169–176 17222557 10.1016/j.knee.2006.11.002

[2] Duncan R, Peat G, Thomas E, Wood L, Hay E, Croft P (2009) Does isolated patellofemoral osteoarthritis matter? Osteoarthr Cartil 17(9):1151–1155 10.1016/j.joca.2009.03.016 19401244

[3] Davies AP, Vince AS, Shepstone L, Donell ST, Glasgow MM (2002) The radiologic prevalence of patellofemoral osteoarthritis. Clin Orthop Relat Res (1976–2007) 402:206–212 10.1097/00003086-200209000-00020 12218486

[4] Kobayashi S, Pappas E, Fransen M, Refshauge K, Simic M (2016) The prevalence of patellofemoral osteoarthritis: a systematic review and meta-analysis. Osteoarthr Cartil 24(10):1697–1707 10.1016/j.joca.2016.05.011 27188684

[5] Van Jonbergen HPW, Poolman RW, van Kampen A (2010) Isolated patellofemoral osteoarthritis: a systematic review of treatment options using the GRADE approach. Acta Orthop 81(2):199–205 20175647 10.3109/17453671003628756 PMC2852157

[6] Rand JA (1994) The patellofemoral joint in total knee arthroplasty. JBJS 76(4):612–620 10.2106/00004623-199404000-00019 8150832

[7] Lonner JH (2007) Patellofemoral arthroplasty. JAAOS-Journal Am Acad Orthop Surg 15(8):495–506 10.5435/00124635-200708000-00006 17664369

[8] Mont MA, Haas S, Mullick T, Hungerford DS (2002) Total knee arthroplasty for patellofemoral arthritis. JBJS 84(11):1977–1981 10.2106/00004623-200211000-00011 12429758

[9] Roussot MA, Haddad FS (2018) The evolution and role of patellofemoral joint arthroplasty: the road less travelled, but not forgotten. Bone Jt Res 7(12):636–638 30662710 10.1302/2046-3758.712.BJR-2018-0303 PMC6318750

[10] Sisto DJ, Sarin VK (2006) Custom patellofemoral arthroplasty of the knee. JBJS 88(7):1475–1480 10.2106/JBJS.E.00382 16818972

[11] Batailler C, Putzeys P, Lacaze F, Vincelot-Chainard C, Fontalis A, Servien E, Lustig S (2023) Patellofemoral arthroplasty is an efficient strategy for isolated patellofemoral osteoarthritis with or without robotic-assisted system. J Personalized Med 13(4):625 10.3390/jpm13040625 PMC10142406 37109011

[12] Pacchiarotti G, Todesca A, Coppola M, Gumina S (2024) Robotic-assisted patellofemoral arthroplasty provides excellent implant survivorship and high patient satisfaction at mid-term follow-up. Int Orthop 48(8):2055–2063 38819666 10.1007/s00264-024-06224-2 PMC11246259

[13] XXXX

[14] van Jonbergen HPW, Werkman DM, Barnaart LF, van Kampen A (2010) Long-term outcomes of patellofemoral arthroplasty. J Arthroplast 25(7):1066–1071 10.1016/j.arth.2009.08.023 20056375

[15] Jones GG, Campi S, von Knoch F, Lunebourg A, London N, Barrett D, Argenson JN (2025) Indications for the addition of a patellofemoral joint arthroplasty following a previous unicondylar knee arthroplasty–a literature review and Delphi consensus. Arch Orthop Trauma Surg 145(1):120 39797997 10.1007/s00402-024-05738-z PMC11724779

[16] Dy CJ, Franco N, Ma Y, Mazumdar M, McCarthy MM, Della G, Valle A (2012) Complications after patello-femoral versus total knee replacement in the treatment of isolated patello-femoral osteoarthritis. A meta-analysis. Knee surgery, sports traumatology, arthroscopy. Official J ESSKA 20(11):2174–2190 10.1007/s00167-011-1677-8 21987361

[17] Elbardesy H, McLeod A, Gul R, Harty J (2022) Midterm results of modern patellofemoral arthroplasty versus total knee arthroplasty for isolated patellofemoral arthritis: systematic review and meta-analysis of comparative studies. Arch Orthop Trauma Surg 142(5):851–859 33825970 10.1007/s00402-021-03882-4

[18] Li C, Li Z, Shi L, Gao F, Sun W (2021) The short-term effectiveness and safety of second-generation patellofemoral arthroplasty and total knee arthroplasty on isolated patellofemoral osteoarthritis: a systematic review and meta-analysis. J Orthop Surg Res 16(1):358 34078392 10.1186/s13018-021-02509-z PMC8171053

[19] Peng G, Liu M, Guan Z, Hou Y, Liu Q, Sun X, Zhu X, Feng W, Zeng J, Zhong Z, Zeng Y (2021) Patellofemoral arthroplasty versus total knee arthroplasty for isolated patellofemoral osteoarthritis: a systematic review and meta-analysis. J Orthop Surg Res 16(1):264. 10.1186/s13018-021-02414-5 33858458 10.1186/s13018-021-02414-5 PMC8048312

[20] Wu Z, Wang N, Zhang J, Lu C, Rong W, Ding X, Lei G (2025) The perioperative resource use and effectiveness of patellofemoral arthroplasty versus total knee arthroplasty: a meta-analysis. Orthopaedic Surg 10.1111/os.70063 PMC12214395 40371973

[21] Granholm A, Alhazzani W, Møller MH (2019) Use of the GRADE approach in systematic reviews and guidelines. Br J Anaesth 123(5):554–559 31558313 10.1016/j.bja.2019.08.015

[22] Page, M. J., Moher, D., Bossuyt, P. M., Boutron, I., Hoffmann, T. C., Mulrow, C.D., McKenzie, J. E. (2021). PRISMA 2020 explanation and elaboration: updated guidance and exemplars for reporting systematic reviews. bmj, 372 10.1136/bmj.n160 PMC8005925 33781993

[23] Fredborg C, Odgaard A, Sørensen J (2020) Patellofemoral arthroplasty is cheaper and more effective in the short term than total knee arthroplasty for isolated patellofemoral osteoarthritis: cost-effectiveness analysis based on a randomized trial. Bone Joint J 102–B(4):449–457 32228074 10.1302/0301-620X.102B4.BJJ-2018-1580.R3

[24] Joseph MN, Achten J, Parsons NR, Costa ML (2020) The PAT randomized clinical trial: total knee arthroplasty versus patellofemoral arthroplasty in patients with severe arthritis of the patellofemoral joint. Bone Jt J 102(3):310–318 32114806 10.1302/0301-620X.102B3.BJJ-2019-0723.R1

[25] Odgaard A, Madsen F, Kristensen PW, Kappel A, Fabrin J (2018) The mark Coventry award: patellofemoral arthroplasty results in better range of movement and early Patient-reported outcomes than TKA. Clin Orthop Relat Res 476(1):87–100 29529622 10.1007/s11999.0000000000000017 PMC5919242

[26] Odgaard A, Kappel A, Madsen F, Kristensen PW, Stephensen S, Attarzadeh AP (2022) Patellofemoral arthroplasty results in better time-weighted patient-reported outcomes after 6 years than TKA: a randomized controlled trial. Clin Orthop Relat Res 480(9):1707–1718 35315804 10.1097/CORR.0000000000002178 PMC9384928

[27] Clement ND, Howard TA, Immelman RJ, MacDonald D, Patton JT, Lawson GM, Burnett R (2019) Patellofemoral arthroplasty versus total knee arthroplasty for patients with patellofemoral osteoarthritis: equal function and satisfaction but higher revision rate for partial arthroplasty at a minimum eight years’ follow-up. Bone Jt J 101–B(1):41–46 30601045 10.1302/0301-620X.101B1.BJJ-2018-0654.R2

[28] Dahm DL, Al-Rayashi W, Dajani K, Shah JP, Levy BA, Stuart MJ (2010) Patellofemoral arthroplasty versus total knee arthroplasty in patients with isolated patellofemoral osteoarthritis. Am J Orthop (Belle Mead, N.J. 39(10):487–491 21290009

[29] Kamikovski I, Dobransky J, Dervin GF (2019) The clinical outcome of patellofemoral arthroplasty vs total knee arthroplasty in patients younger than 55 years. J Arthroplast 34(12):2914–2917 10.1016/j.arth.2019.07.016 31500912

[30] Lin W, Dai Y, Dong C, Piao K, Hao K, Wang F (2021) Joint awareness after patellofemoral arthroplasty evaluated with the forgotten joint score: a comparison study. Orthop Surg 13(3):833–839 33749150 10.1111/os.12921 PMC8126918

[31] Serino J 3rd, Weintraub MT, Burnett RA 3rd, Angotti ML, Courtney PM, Della Valle CJ (2023) Complications and costs of patellofemoral arthroplasty versus total knee arthroplasty. Knee 41:58–65 36638704 10.1016/j.knee.2022.12.016

[32] Vella-Baldacchino M, Bottle A, Cobb J, Liddle AD (2025) Outcomes of patellofemoral joint arthroplasty compared with total knee arthroplasty for osteoarthritis: a population-based cohort study using data from the National joint registry and hospital episode statistics for England. Bone Jt J 107–B(5):514–521 40306698 10.1302/0301-620X.107B5.BJJ-2024-1273.R2

[33] Omenås HN, Lindalen E, Furnes ON, Fenstad AM, Badawy M (2025) Patellofemoral arthroplasty—patient demographics and revision causes compared with total and medial unicompartmental knee arthroplasty: Long-term follow-up data from the Norwegian arthroplasty register. Acta Orthop 96(6):671–676 40891929 10.2340/17453674.2025.44593 PMC12404100

[34] Hariri, M., Schwab, H., Koch, K. A., Mick, P., Nees, T., Weishorn, J., Reiner,T. (2025) High survivorship and excellent functional outcome in third-generation patellofemoral arthroplasty. J Exp Orthop *12*(2), e70287 10.1002/jeo2.70287 PMC12167627 40521296

[35] Bond EC, Reinke EK, Zirbes C, Poehlein E, Green CL, Danilkowicz RM, Amendola A (2025) Outcomes after patellofemoral arthroplasty with the arthrex iBalance—A third generation implant. Arthroplast Today 33:101666 40226788 10.1016/j.artd.2025.101666 PMC11992531

[36] Vella-Baldacchino M, Davies AR, Bottle A, Cobb J, Liddle AD (2025) Association between surgeon volume and patient outcomes after elective patellofemoral arthroplasty: a population-based cohort study using data from the National joint registry and hospital episode statistics for England. J Bone Jt Surg Am 107(8):819–828 39899649 10.2106/JBJS.24.00703

[37] Villa JC, Paoli AR, Nelson-Williams HW, Badr RN, Harper KD (2021) Onlay patellofemoral arthroplasty in patients with isolated patellofemoral arthritis: a systematic review. J Arthroplast 36(7):2642–2649 10.1016/j.arth.2021.02.054 33795175

[38] Pagano A, Agostinone P, Alesi D, Caputo D, Neri MP, Grassi A, Zaffagnini S (2024) Almost 79% survival rate at 10-year follow‐up for the patellofemoral joint arthroplasty: an Italian prosthetic registry study. Knee Surg Sports Traumatol Arthrosc 32(6):1525–1530 38529690 10.1002/ksa.12150

[39] Ziedas A, Miller A, Biddle E, Laker M, Michaelson J, Frush T, Markel DC (2025) Manual vs robotic patellofemoral arthroplasty outcomes: a Michigan arthroplasty registry collaborative quality Initiative-Based study. J Orthop 10.1016/j.jor.2025.05.002 PMC12181901 40548200

[40] Saidy E, Du P, Cuthbert AR, Lewis PL, Leie M (2025) Can robotic-assisted arthroplasty change the high early revision rate after patello-femoral arthroplasty? An Analysis From the Australian Orthopaedic Association National Joint Replacement Registry. The J Arthroplast 10.1016/j.arth.2025.06.035 40513908

[41] Arteaga J, Poblete E, Martin F, Domecq G, Figueroa D (2025) Return to sports and recreational activities after patellofemoral arthroplasty: a systematic review. J ISAKOS 14:100925 40633908 10.1016/j.jisako.2025.100925

[42] Tarassoli P, Punwar S, Khan W, Johnstone D (2012) Patellofemoral arthroplasty: a systematic review of the literature. Open Orthop J 6:340–347. 10.2174/1874325001206010340 22927894 10.2174/1874325001206010340 PMC3419872

[43] Chawla H, Nwachukwu BU, Van Der List JP, Eggman AA, Pearle AD, Ghomrawi HM (2017) Cost effectiveness of patellofemoral versus total knee arthroplasty in younger patients. Bone Jt J 99(8):1028–1036 28768779 10.1302/0301-620X.99B8.BJJ-2016-1032.R1

[44] Rasmussen LE, Hoffmann AG, Blanche P, Espersen F, Justesen TF, Rasmussen LE, Hangaard S, Christensen R, Odgaard A (2025) Surgeon training and revision rates after patellofemoral arthroplasty. JAMA Netw Open 8(6): e2517825 10.1001/jamanetworkopen.2025.17825 PMC12205405 40577013

[45] Marullo M, Bargagliotti M, Russo A, Zero E, Romagnoli S (2025) Impact of age on patellofemoral arthroplasty Outcomes, osteoarthritis Progression, and survivorship: the youngest and oldest achieve the best results. J Arthroplast 10.1016/j.arth.2025.06.064 40550375

[46] Leadbetter WB, Seyler TM, Ragland PS, Mont MA (2006) Indications, contraindications, and pitfalls of patellofemoral arthroplasty. JBJS 88(suppl4):122–137 10.2106/JBJS.F.00856 17142442

[47] Treu EA, Frandsen JJ, Al Saidi NN, Blackburn BE, Pelt CE, Anderson LA, Gililland JM (2023) Outcomes are compromised when revising patellofemoral arthroplasties for patellar component failures. J Arthroplast 38(7 Suppl 2):S369–S375 10.1016/j.arth.2023.02.083 36889525

