# Supplementary material for: Patellofemoral vs. total knee arthroplasty for isolated patellofemoral osteoarthritis: evidence-based recommendations from a systematic review with GRADE assessment
Source: Arch Orthop Trauma Surg. 2026 Feb 12;146(1):61. doi: 10.1007/s00402-026-06217-3 (PMC12901212; doi:10.1007/s00402-026-06217-3)
Supplement: Supplementary file 1 — Supplementary Material 1 [file 402_2026_6217_MOESM1_ESM.docx]

**Supplementary Table 1. Search strategy.**

| **Database** | **Search Strategy** |  |
| --- | --- | --- |
| **PubMed** | (("patellofemoral arthroplasty"[Title/Abstract] OR "patellofemoral replacement"[Title/Abstract] OR "total knee arthroplasty"[Title/Abstract] OR "total knee replacement"[Title/Abstract]) OR ("Patellofemoral Arthroplasty"[Mesh] OR "Knee Prosthesis"[Mesh] OR "Arthroplasty, Replacement, Knee"[Mesh])) AND ("patellofemoral osteoarthritis"[Title/Abstract] OR "patellofemoral arthritis"[Title/Abstract] OR "patellofemoral arthrosis"[Title/Abstract] OR ("Osteoarthritis"[Mesh] AND "Patellofemoral Joint"[Mesh])) | 393 |
| **Cochrane Library** | ("patellofemoral arthroplasty" OR "patellofemoral replacement" OR "total knee arthroplasty" OR "total knee replacement") AND ("patellofemoral osteoarthritis" OR "patellofemoral arthritis" OR "patellofemoral arthrosis") | 19 |
| **Google Scholar** | ("patellofemoral arthroplasty" OR "patellofemoral replacement" AND "total knee arthroplasty" OR "total knee replacement")  AND  ("patellofemoral osteoarthritis" OR "patellofemoral arthritis" AND "patellofemoral arthrosis") | 100 |
| **Total** | 474 after duplicated removal | 512 |

**Supplementary Table 2. PICO Framework** summarizes the Population, Intervention, Comparator, and Outcomes (PICO) criteria for the systematic review, structured in accordance with PRISMA.

| **Element** | **Definition** |
| --- | --- |
| Population (P) | Adults with radiologically and/or clinically confirmed isolated patellofemoral osteoarthritis (PFOA). |
| Intervention (I) | Patellofemoral arthroplasty (PFA) — any prosthetic design. |
| Comparator (C) | Total knee arthroplasty (TKA) with patellar resurfacing. |
| Outcomes (O) | **Primary outcomes:**  1. Improvement in validated PROMs.   2. Implant survival at two, five, and ten years.   **Secondary outcomes:**  3. Complications (surgical and medical)  4. Patient Satisfaction   5. Return to sport   6. Cost-effectiveness |

**Supplementary Table 3. RoB 2 assessment.**

| **Study** | **D1** | **D2** | **D3** | **D4** | **D5** | **Overall** |
| --- | --- | --- | --- | --- | --- | --- |
| Odgaard et al. 2018 | Low | Low | Low | Low | Low | Low |
| Odgaard et al. 2022 | Low | Low | Some concerns | Low | Low | Some concerns |
| Fredborg et al. 2020 | Low | Low | Low | Low | Some concerns | Low |
| Joseph et al. 2020 | Some concerns | Low | Low | Low | Some concerns | Some concerns |

**Supplementary Figure 1. RoB 2 visualization for RCTs.**


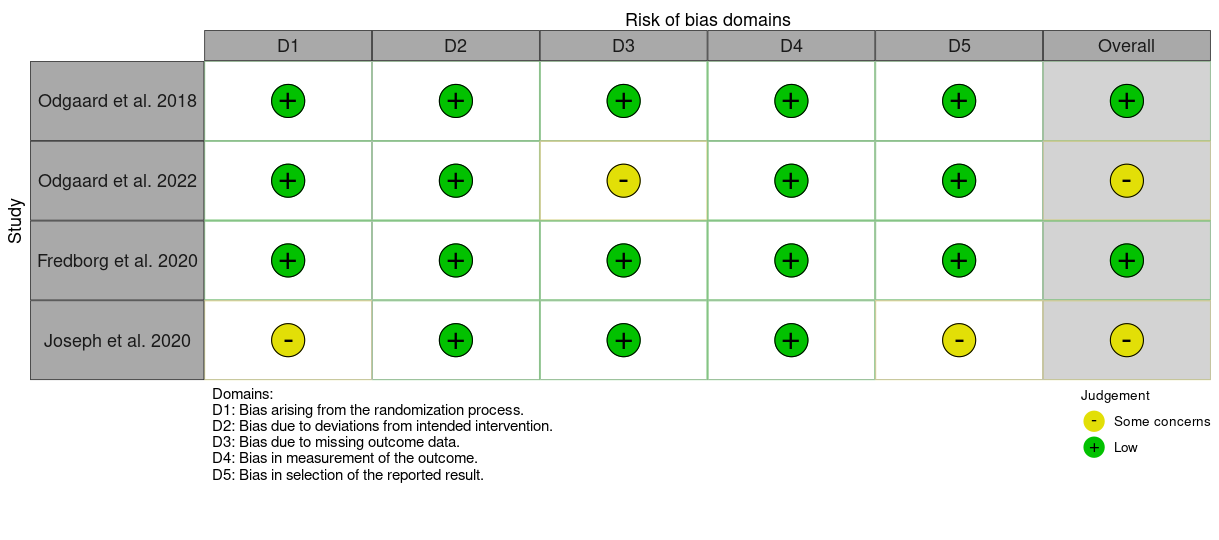


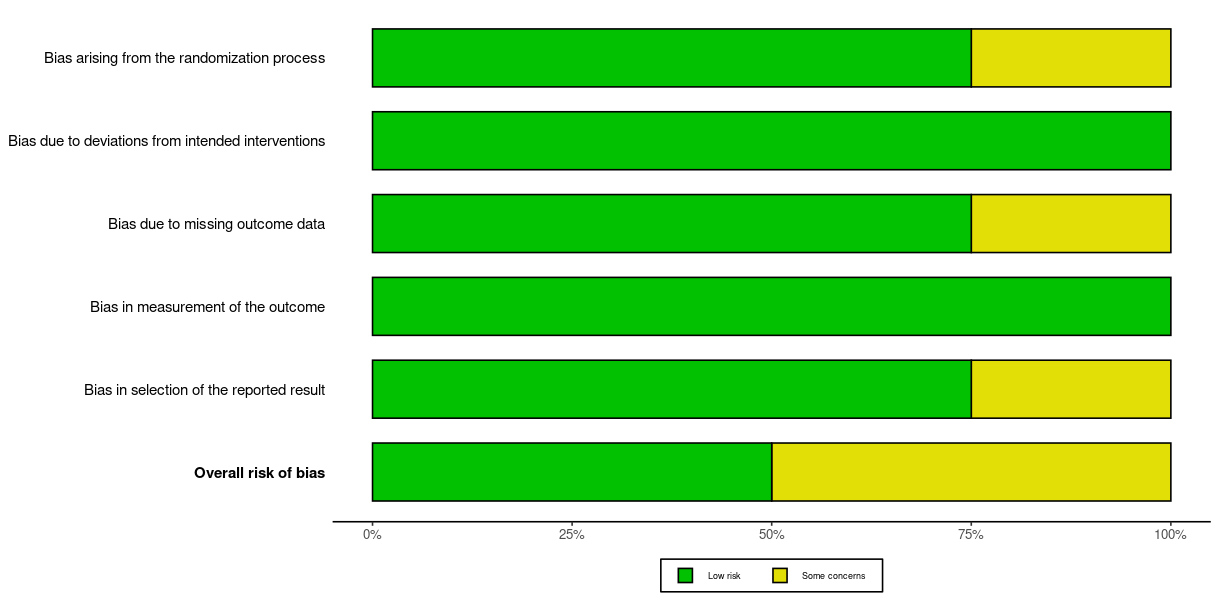


**Supplementary Table 4. ROBINS-I assessment for observational studies.**

| **Study** | **D1** | **D2** | **D3** | **D4** | **D5** | **D6** | **D7** | **Overall** |
| --- | --- | --- | --- | --- | --- | --- | --- | --- |
| Vella-Baldacchino et al. 2025 | Moderate | Low | Low | Low | Low | Moderate | Moderate | Moderate |
| Serino et al. 2023 | Moderate | Moderate | Low | Low | Low | Moderate | Moderate | Moderate |
| Clement et. al 2019 | Moderate | Moderate | Low | Low | Low | Moderate | Moderate | Moderate |
| Dahm et al. 2010 | Moderate | Serious | Low | Low | Moderate | Moderate | Moderate | Serious |
| Kamikovski et al. 2019 | Moderate | Moderate | Low | Low | Low | Moderate | Moderate | Moderate |
| Lin et al. 2021 | Moderate | Serious | Low | Low | Moderate | Serious | Moderate | Serious |

**Supplementary Figure 2. ROBINS-I assessment for observational studies.**


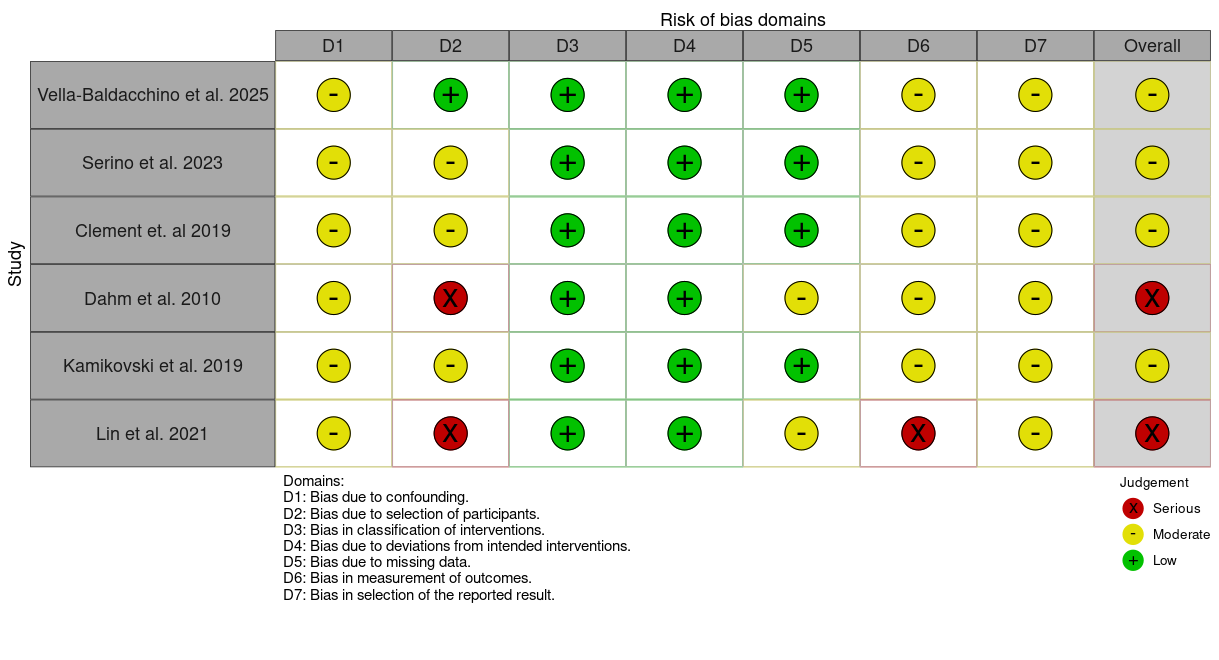


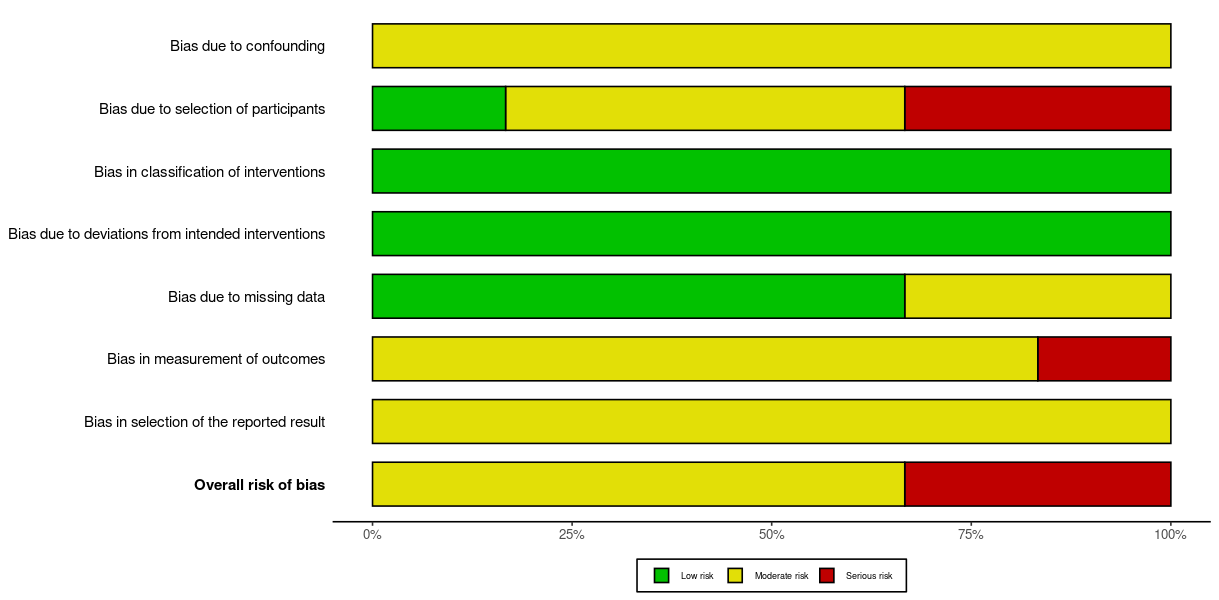


**Supplementary Table 5. Clinical and Methodological Heterogeneity of Included Studies Precluding Valid Quantitative Meta‑analysis.**

| **Study** | **Design / data source** | **Primary outcome(s)** | **Follow‑up (years)** | **Key sources of heterogeneity vs other studies** |
| --- | --- | --- | --- | --- |
| Fredborg 2020 | RCT | Cost‑effectiveness (QALY, EQ‑5D, SF‑6D) | 2 | Economic primary endpoint; different outcome metric (QALY) and effect scale; short term follow‑up. ​ |
| Joseph 2020 | Pragmatic RCT | WOMAC | 5 | Multiple implant designs, routine‑practice setting; PROMs only; no revisions reported; different PROM compared to other RCTs. ​ |
| Odgaard 2018 | RCT | SF‑36 pain | 2 | Different PROMs (SF‑36/KOOS/OKS), short follow‑up, underpowered for revisions. ​ |
| Odgaard 2022 | RCT (extension) | Time‑weighted PROMs (AUC 0–6 yrs) SF‑36 | 6 | Uses time‑weighted AUC of PROMs (SF‑36/KOOS/OKS) rather than point estimates; limited revision events; distinct statistical approach. ​ |
| Clement 2019 | Propensity‑matched cohort | OKS | 8–15 (mean 9.2) | Observational design; older population; long‑term survival focus; different confounding structure. ​ |
| Dahm 2010 | Retrospective cohort | KSS, UCLA, Tegner | ≈2–2.5 | Very small sample; different PROMs (KSS, activity scores); no revisions reported. ​ |
| Kamikovski 2019 | Case‑matched cohort (<55 yrs) | KOOS, WOMAC | 2 | Younger, high‑activity population; small sample; different PROM set; underpowered for rare events. ​ |
| Lin 2021 | Case‑matched cohort | FJS, KSS | 1 and 3 | Different primary PROM (FJS), Asian population, short follow‑up; no revisions reported. ​ |
| Serino 2023 | Administrative registry (USA) | Costs, complications, revisions | 5 | Claims‑based data; large, heterogeneous US population; different coding and confounding; survival expressed as KM curves. ​ |
| Vella‑Baldacchino 2025 | National registry (UK NJR‑HES) | 30‑day complications, 10‑yr survival | 10 | Massive sample, different health system; outcomes limited to complications and revisions; broader OA indications. |
